# Development and Validation of a 15-Item Japanese Health Knowledge Test

**DOI:** 10.2188/jea.JE20090096

**Published:** 2010-07-05

**Authors:** Yasuharu Tokuda, Tomoya Okubo, Haruo Yanai, Nobutaka Doba, Michael K. Paasche-Orlow

**Affiliations:** 1 Institute of Clinical Medicine, Graduate School of Comprehensive Human Sciences, University of Tsukuba, Tsukuba, Ibaraki, Japan; 2 The National Center for University Entrance Examinations, Tokyo, Japan; 3 St Luke’s Graduate School of Nursing, Tokyo, Japan; 4 The Life Planning Center, Tokyo, Japan; 5 Section of General Internal Medicine, Department of Medicine, Boston University School of Medicine, Boston, USA

**Keywords:** health knowledge, health literacy, socioeconomic status

## Abstract

**Background:**

Health literacy affects the acquisition of health knowledge and is thus linked to health outcomes. However, few scales have been developed to assess the level of health knowledge among the general public.

**Methods:**

The 15-item Japanese Health Knowledge Test (J-HKT) was developed by using item response theory to score an item pool. We examined the construct validity of the J-HKT in relation to health literacy items, and analyzed the sociodemographic and behavioral factors associated with poor health knowledge.

**Results:**

We enrolled 1040 adult participants (mean age, 57 years; women, 52%). The 15 items that best identified people with poor health knowledge were selected. For all items on the J-HKT, the information function curves had a peak in the negative spectrum of the latent trait. As compared with participants reporting high levels of income, educational attainment, and literacy, those with low levels of income, education, and literacy had a lower total score on the J-HKT. As compared with non/light drinkers, moderate and heavy drinkers had lower total scores on the J-HKT.

**Conclusions:**

The J-HKT may prove useful in measuring health knowledge among the general public, and in identifying and characterizing those with poor health knowledge.

## INTRODUCTION

A growing body of evidence supports the impact of low health literacy on the health of individuals^1^; therefore, recent attention has focused on the elucidation of potential causal pathways linking low health literacy to poor health.^2^^,^^3^ Among the mechanisms that mediate the influence of health literacy on the health of individuals, the effect of health literacy on health knowledge may be one of the most consistent and critical factors.^4^^–^^6^ It has been proposed that low health literacy leads to poor health knowledge and, ultimately, to worse health outcomes, because people with low health literacy have difficulty in acquiring the health knowledge necessary to navigate the healthcare system and to practice effective self-care.

Patients with poor knowledge of illness prevention and chronic diseases have lower adherence to medical instructions and are more likely to have high-risk health behaviors.^7^^–^^10^ Thus, these individuals are less likely to utilize healthcare services, such as recommended vaccination and health screening programs.^11^^–^^13^ In addition, during both acute and chronic illnesses, the quality of self-care is poor among those with limited knowledge, which may manifest in the greater use of potentially harmful complementary or alternative medicine.^14^

Many studies have evaluated the relationship between health literacy and health knowledge.^4^^,^^5^^,^^7^^,^^8^^,^^15^^,^^16^ These have mostly focused on patients with specific illnesses, such as asthma, diabetes, congestive heart failure, hypertension, and human immunodeficiency virus infection; few have evaluated the association between low health literacy and poor health knowledge in the general public. In patients with chronic diseases, the relationship between health literacy and health knowledge of a particular disease has been confirmed.^4^^,^^5^^,^^7^^,^^8^^,^^15^^,^^16^ In order to better understand the relationship between health literacy and health knowledge, and to help target education and guide disease prevention for the general public, it would be useful to examine the relationship between health knowledge and health literacy in the general public. However, this objective cannot be realized without a tool to assess general health knowledge. Such a tool would be particularly useful if it were short, if it could differentiate among people at the low end of the health knowledge spectrum, and if it could be administered in a mode other than in-person interview. Thus, in the present study, we used nominal categories modeling of item response theory (IRT) analysis to develop a test of general health knowledge for Japanese adults. To evaluate construct validity, we examined the association between this health knowledge test and health literacy. In addition, we identified the sociodemographic and health behavioral factors that were significantly associated with poor health knowledge.

## METHODS

### Study participants

The data for this study were collected from responses to a national cross-sectional online survey conducted from 3 July to 8 July 2008. Institutional review board approval was obtained from the National Institute of Japanese Language. Japan was divided into 10 regions: Hokkaido, Tohoku, Kanto, Tokai, Keihin, Hokuriku, Kyouhanshin, Chugoku, Shikoku, and Kyushu. The number of potential participants was determined within each region from a panel of people registered by Yahoo JAPAN Co. (Tokyo, Japan) by means of probability sampling proportionate to age and sex, using Japanese national census data of population distributions for people aged 30–90 years in 2007. People younger than 30 years were excluded because our aims included evaluation of the potential association between health knowledge and final educational attainment. In addition, health care workers, such as physicians, nurses, hospital workers, and public health workers, were excluded. No gifts or payments were given for participating in the survey.

### Data collection

The survey gathered demographic and socioeconomic data, as well as responses to the questionnaire for health literacy and the test of health knowledge. Demographic data included age, sex, annual income, education, and occupation. Regarding annual income, cutoff points of 2, 4, 6, and 8 million Japanese Yen (JY) were used to generate 5 income categories (the average exchange rate for 1 US dollar in July 2008 was about 100 JY). We used these income cutoffs because the National Tax Agency regards an income of 2 million JY as the cutoff level for low-wage workers and reports the income distribution in this fashion. For educational attainment, 5 categories were used (did not graduate high school, high school graduate, vocational school, short-term college, and undergraduate/postgraduate degree). For occupational status, 5 categorical levels were included: working full-time, homemaker, working part-time, retired, and not currently working. Survey items also assessed current and past smoking, current alcohol use, and chronic conditions (cancer, cardiovascular disease, hypertension, diabetes, arthritis, asthma or chronic obstructive pulmonary disease, and depression), as previously described.^2^

Current alcohol consumption was categorized into 3 categories: non/light, moderate, and heavy. Non/light drinkers were defined as those who drank less than once a week; moderate and heavy users included those who drank at least once a week. In addition, heavy users were defined as those who drank in a day ≥3 glasses of beer, ≥540 ml of Japanese sake (*nihonshu*), three-quarters of a bottle or more of wine, or ≥180 ml of whisky. All remaining participants were defined as moderate users.

Health literacy was measured by self-report using 2 validated screening questions.^17^^,^^18^ Specifically, we asked: “How often do you have problems learning about your medical condition because of difficulty understanding written information?” (Item 1: “Problems learning”) and “How often do you have someone help you read hospital materials?” (Item 2: “Help reading”). The 5-point Likert response scale was, “Never”, “Occasionally”, “Sometimes”, “Often”, or “Always”. These 2 items have been shown to predict scores on commonly used English-language measures of health literacy: the Short Test of Functional Health Literacy in Adults (STOFHLA) and the Rapid Estimate of Adult Literacy in Medicine (REALM).^17^^,^^18^ Due to the linguistic differences between English and Japanese, English-language instruments for measuring health literacy cannot be simply translated. Thus, we used these 2 self-report items as surrogate measures of health literacy.^17^^–^^19^

### Development of the Japanese Health Knowledge Test (J-HKT)

The first phase of development included item generation by a group of experts in healthcare, literacy, linguistics, and mass media. This 25-member group included physicians, nurses, pharmacists, linguists, journalists, university researchers in communication, and representatives of patient advocate groups. Each item was developed with a single correct response among a list of 4 choices. When providing the item test to the study participants, they were advised that there was a 2-minute time limit for each item. Each item was scored as correct or incorrect.

In the second phase of development, the 48-item pool was shortened using item response theory (IRT) analysis, specifically the nominal categories model. This model was proposed by Bock^20^ as an extension of IRT analysis for nominally scored items. As compared with the use of a graded categories model or a binary logistic model, the nominal categories model is more effective in examining the full spectrum of contributions for each item and the possible responses in an instrument. For this purpose, we used a sample size large enough to meet the requirements of nominal categories modeling.

In the nominal categories model, the response probability *p_ijk_* that respondent *i* with a latent trait θ*_i_* response to category *k* (*k* = 1, 2,…, *K_j_*) of item *j* is described as follows^21^:

$$p_{ijk}=\frac{\exp(\alpha_{jk}\theta_{i}+\gamma_{jk})}{\sum_{k^{\prime }=1}^{K_{j}}\exp(\alpha_{jk^{\prime }}\theta_{i}+\gamma_{jk^{\prime }})}$$

where *K_j_* denotes the number of the category of item *j*. We cannot interpret the parameters of the categories independently in the nominal categories model because the equation defined for a response probability to the category contains other parameters. Thus, in order to estimate item parameters, Okubo suggested that a restriction be imposed as follows^22^:

$$\alpha_{j1}=\gamma_{j1}=0$$

The role of the alpha parameter is that of a slope in the linear function. A larger slope implies that the item clearly discriminates the latent trait θ*_i_*, while a smaller slope implies low discrimination. The role of the gamma parameter is that of an intercept. A larger intercept gamma suggests that the item is difficult to solve, while a smaller intercept gamma suggests it is easy to solve.

Next, the item response category characteristic curve (IRCCC) is determined by the relative relations among parameters; thus, each parameter cannot be interpreted alone. The usual method to analyze the characteristics of items is to draw the IRCCC by using the estimated parameters. The IRCCC is a multinomial logistic regression curve whose independent variable is a factor—in this case, health knowledge.

Item information functions were then generated for each item. Item information function curves were derived from the response probabilities from the IRCCCs. The standard error of measurement curve can be calculated as the reciprocal of the square root of the item information function. Item information functions describe responses at different levels of a latent trait—health knowledge in this study. A combination of all items together was used to generate the test information function, and an item reduction procedure was performed based on the item information functions. Participants with a score that was ≥1 standard deviation lower than the mean were classified as having a low score.

Phase 3 of development sought to support the validity of the J-HKT. The face validity of the J-HKT was confirmed by the aforementioned expert panel. Next, for construct validity, we hypothesized that health literacy would be associated with improved J-HKT scores and thus the association between literacy and J-HKT scores was examined by using the nonparametric test for trend across ordered groups developed by Cuzick.^23^

Associations between sociodemographic characteristics and J-HKT scores were evaluated by a logistic regression model that included age and sex, as well as additional variables found to be significant in univariate analyses. Statistical analyses were performed using R version 2.6.6 (R Foundation for Statistical Computing) and STATA 10.0 (College Station, Texas, USA), and graphics were generated using Mathematica version 6.0 (Wolfram Research, Illinois, USA). A 2-tailed *P* value <0.05 was considered statistically significant.

## RESULTS

Of 2500 subjects randomly selected from the online panel, 1074 participated in the study (response rate, 43.0%). Among these, after deleting data from participants working in the health care industry, data for 1040 persons were available for our analysis and were considered as the final sample. Table 1 shows the sociodemographic characteristics of all participants; 52% were women and the mean age was 57 years (range, 30–90).

**Table 1.** Characteristics of participants (*n* = 1040)

Characteristic	Mean (SD) or *n*, %
Age (years)	57 (15)
Sex	
Male	497, 48%
Female	543, 52%
Income (Japanese Yen)	
<2 million	92, 9%
2–3.99 million	264, 25%
4–5.99 million	290, 28%
6–7.99 million	160, 15%
8 million or more	234, 23%
Education	
<Grade 12	51, 5%
High school graduate	379, 36%
Vocational school	107, 10%
Some college	139, 13%
University or graduate degree	364, 35%
Working status	
Working full-time	445, 43%
Homemaker	273, 26%
Working part-time	91, 9%
Retired	135, 13%
Currently not working	96, 9%

Smoking	
Current	200, 19%
Former	247, 24%
Never	593, 57%
Current alcohol use	
None/light	588, 57%
Moderate	407, 39%
Heavy	45, 4%

Chronic condition	
Cancer	38, 4%
Cardiovascular disease	21, 2%
Hypertension	221, 21%
Diabetes	55, 5%
Arthritis	45, 4%
Asthma or COPD	29, 3%
Depression	33, 3%

COPD = chronic obstructive pulmonary disease.

The initial item pool contained 48 items that covered knowledge of body parts, diseases, hospitals, drugs, healthcare systems, health policy, and home care. The expert panel considered these 48 items to have adequate content validity, and to represent the range of patient knowledge required to understand common medical problems. Based on the item information functions of the IRT analysis for health knowledge testing in the 1040 participants, a 15-item J-HKT was produced from the initial 48-item pool (Table 2 and Supplement).

**Table 2.** Estimated parameters for the 15 items of the Japanese Health Knowledge Test

Item number	Alpha (slope parameter)				Gamma (location parameter)			
	Category				Category			
	
	1	2	3	4	1	2	3	4
1	0.00	0.07	0.46	0.36	0.00	0.88	2.04	−0.41
2	0.00	0.62	1.56	0.91	0.00	0.77	2.99	2.06
3	0.00	−1.53	−0.30	−0.45	0.00	−2.25	0.13	0.64
4	0.00	−0.69	0.33	−0.47	0.00	1.26	2.04	0.21
5	0.00	2.70	2.19	1.49	0.00	4.65	2.58	1.93
6	0.00	1.67	0.94	2.26	0.00	3.28	0.42	0.64
7	0.00	0.63	−1.79	−2.02	0.00	0.71	−2.68	−3.15
8	0.00	0.26	0.23	1.02	0.00	−0.16	1.08	2.25
9	0.00	−1.07	−0.87	−1.04	0.00	−2.04	−2.50	−2.13
10	NA	0.00	1.53	NA	NA	0.00	3.54	NA
11	0.00	0.56	NA	0.32	0.00	2.53	NA	0.69
12	0.00	−0.74	−0.87	0.24	0.00	−1.42	−0.71	−0.17
13	0.00	−1.52	0.96	NA	0.00	−2.56	0.88	NA
14	0.00	0.81	−0.33	−0.25	0.00	1.37	−0.50	−0.06
15	0.00	−1.18	−0.21	0.69	0.00	−0.91	−0.34	1.12

Values for category 1 were set to 0 for estimating parameters.

NA, not available.

**Figure 1.**
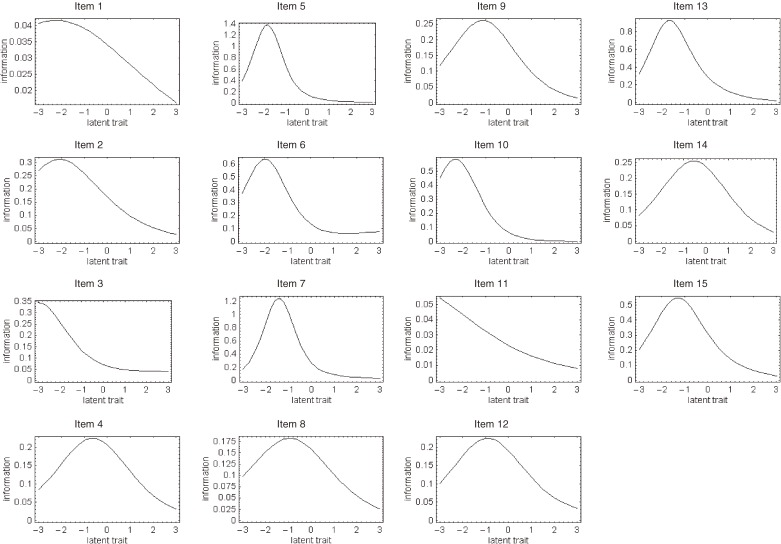
Item information function curves for each item of the Japanese Health Knowledge Test were generated by analysis of data from 1040 Japanese adults. The curves were derived from the response probabilities from the item response category characteristic curves. The standard error of measurement curve was calculated as the reciprocal of the square root of the item information function. Note: the scales for the y-axis differ among items.

Regarding each response to individual items of the J-HKT, all IRCCCs of the J-HKT satisfied the assumption of monotonicity, ie, scores for each item were higher among participants with a higher overall J-HKT score. For most items, a greater number of intersections of probability curves of item responses was shifted to the negative spectrum of the latent trait. Figure 1 shows the item information function for individual items of the J-HKT.

To better discriminate between people with poor health knowledge and those with intermediate or higher levels of health knowledge, 15 items with the highest information function at −0.85 (those with the lowest percentile of 20% of overall scores in all participants) of latent trait θ*_i_* were included in the J-HKT. Thus, we chose items able to differentiate among people at the low end of health knowledge; as such, the curves for all items of the J-HKT show a peak of the information functions in the negative spectrum of the latent trait.

**Figure 2.**
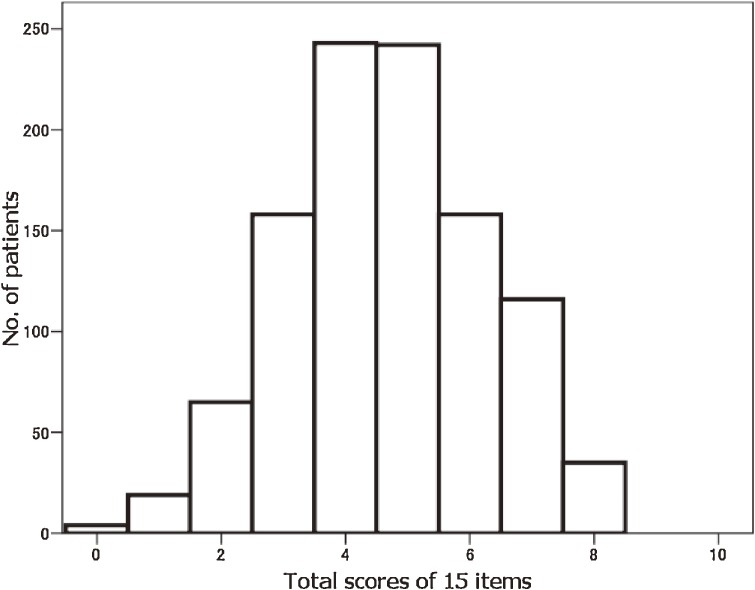
Histogram of total scores on the Japanese Health Knowledge Test.

**Figure 3.**
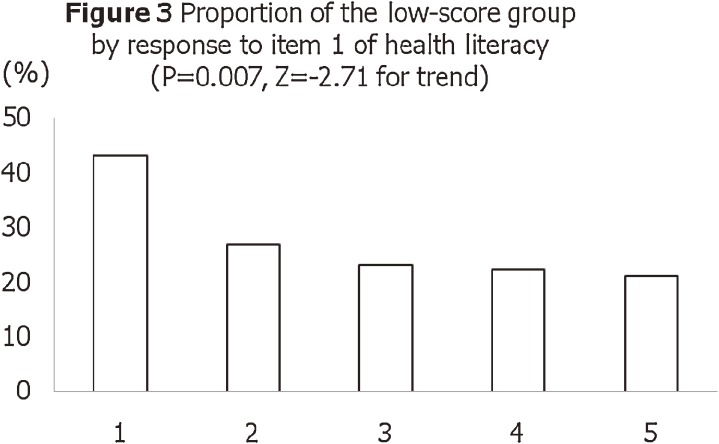
Proportion of participants with poor health knowledge, by response to item 1 (“Problems learning”) on the health literacy test. The question was, “How often do you have problems learning about your medical condition because of difficulty understanding written information?” The 5-point Likert response scale was, “Always” (1), “Often” (2), “Sometimes” (3), “Occasionally” (4), and “Never” (5). Participants with lower literacy represented a higher proportion of those with a low score on the Japanese Health Knowledge Test.

**Figure 4.**
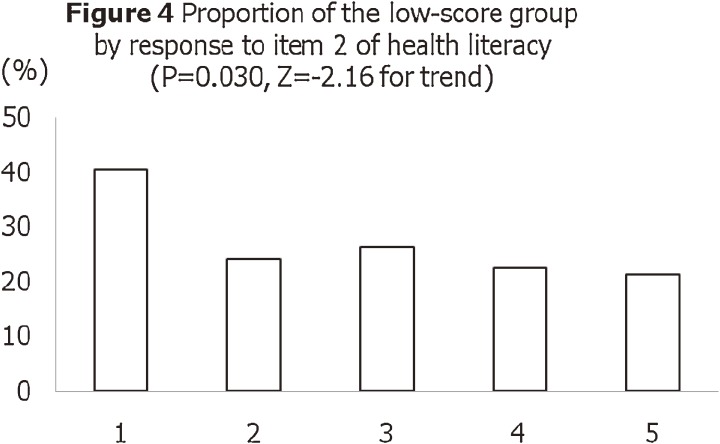
Proportion of participants with poor health knowledge, by response to item 2 (“Help reading”) on the health literacy test. The question was, “How often do you have someone help you read hospital materials?” The 5-point Likert response scale was, “Always” (1), “Often” (2), “Sometimes” (3), “Occasionally” (4), or “Never” (5). Participants with lower literacy represented a higher proportion of those with a low score on the Japanese Health Knowledge Test.

**Figure 5.**
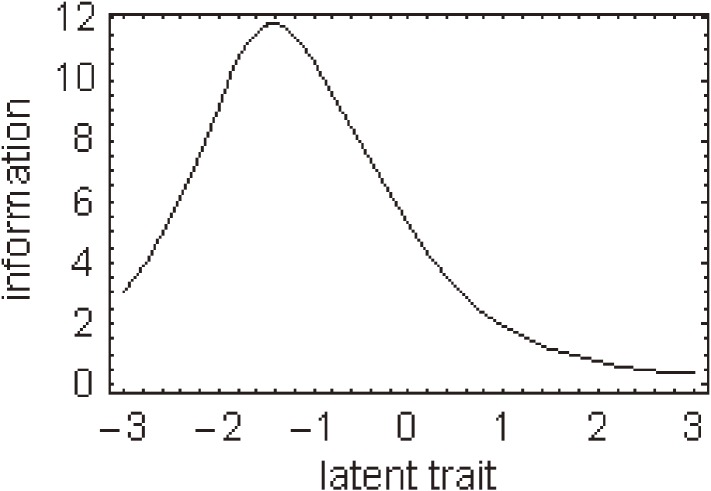
Item information function curve for the 15-item Japanese Health Knowledge Test.

**Figure 6.**
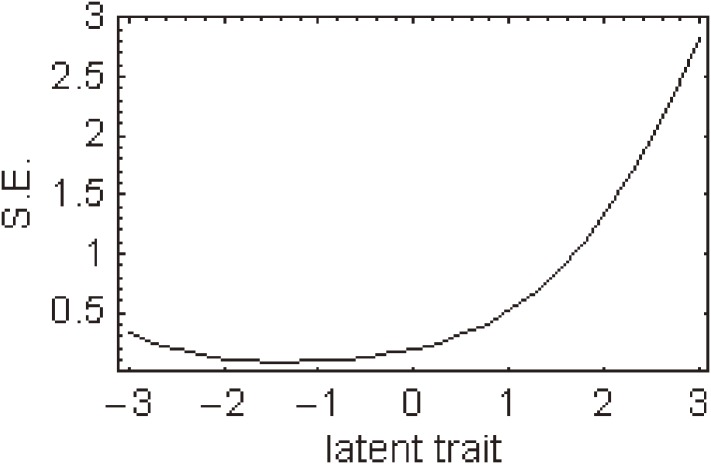
Standard error of the item information function for the 15-item Japanese Health Knowledge Test. S.E. indicates standard error.

Figure 2 shows a histogram of total scores for the 15-item J-HKT. The mean score was 4.7 and the standard deviation was 1.6; the median score was 5.0 and the mode was 4.0. The score is normally distributed, with a skewness of −0.37 and a kurtosis of −0.38. Figures 3 and 4 show the proportions of participants with poor health knowledge, by responses to the 2 health literacy items (“Problems learning” and “Help reading”). There were statistically significant associations between responses to the health literacy items and total score on the J-HKT (ie, construct validity). Figure 5 shows the item information function curve of the 15-item J-HKT, and Figure 6 depicts the standard error curve of the item information function of the 15-item J-HKT (the standard error is the reciprocal of the item information function).

Table 3 shows the distributions of total score on the J-HKT by sociodemographic characteristics, smoking, and alcohol use. Overall, 36% of participants had a score of 0–3, which was defined as poor health knowledge (ie, more than 1 standard deviation below the mean, 4.7 − 1.6 = 3.1). Age, sex, and employment status were not associated with test scores; however, participants with low income and low educational attainment were more likely to have a lower score on the J-HKT. Although smoking status was not associated with J-HKT score, those with higher current alcohol use had a lower total score on the J-HKT.

**Table 3.** Score results of the 15-item Japanese Health Knowledge Test (*n* = 1040)

Characteristic	Total score			Group with low score^a^	
	
	Mean	SD	*P*-value	*n* (%)	*P*-value
Age (years)					
<65 (*n* = 685)	4.67	1.63	0.660^d^	162 (24)	0.996^b^
≧65 (*n* = 355)	4.63	1.57	(0.441)	84 (24)	(0.001)
Sex					
Male	4.62	1.63	0.455^d^	125 (25)	0.277^b^
Female	4.69	1.58	(0.748)	121 (22)	(1.181)
Income (Japanese Yen)					
<2 million	4.17	1.46	<0.001^d^	27 (29)	0.004^c^
2–3.99 million	4.55	1.60	(4.150)	72 (27)	(−2.89)
4–5.99 million	4.63	1.66		72 (25)	
6–7.99 million	4.73	1.54		32 (20)	
8 million or more	4.96	1.58		43 (18)	
Education					
<Grade 12	4.24	1.59	<0.001^d^	17 (33)	0.021^c^
High school graduate	4.50	1.55	(4.340)	98 (26)	(−2.31)
Vocational school	4.37	1.31		26 (24)	
Some college	4.78	1.66		31 (22)	
University or graduate ​ degree	4.92	1.68		74 (20)	
Working status					
Working full-time	4.67	1.63	0.565^e^	110 (25)	0.938^b^
Homemaker	4.74	1.66	(0.740)	61 (22)	(0.805)
Working part-time	4.51	1.49		21 (23)	
Retired	4.71	1.55		30 (22)	
Currently not working	4.47	1.51		24 (25)	

Smoking					
Current	4.49	1.59	0.154^e^	56 (28)	0.079^b^
Former	4.79	1.53	(1.880)	47 (19)	(5.090)
Never	4.66	1.64		143 (24)	
Current alcohol use					
None/light	4.74	1.59	0.027^d^	121 (21)	0.004^c^
Moderate	4.57	1.61	(2.210)	109 (27)	(2.850)
Heavy	4.31	1.72		16 (36)	

SD = standard deviation.

^a^Participants with a score of 0–3 points, ie, mean − SD.

^b^The chi-square test was used. The numbers in parentheses are the chi-square statistic.

^c^The trend test was used. The numbers in parentheses are the z-statistic.

^d^Linear regression was used. The numbers in parentheses are the t-statistic.

^e^ANOVA was used. The numbers in parentheses are the F-statistic.

Table 4 presents the results of the logistic regression model for poor health knowledge on the J-HKT (0–3, yes versus no) adjusted for age, sex, income, education, and current alcohol use. Compared with those with an income >8 million JY, those with income ≥2 and <4 million JY were more likely to have poor health knowledge (odds ratio [OR], 1.68; 95% confidence interval [CI], 1.08–2.62) and those with an income <2 million JY were also more likely to have poor health knowledge (1.84; 1.02–3.31). In addition, as compared with university degree holders, those who had not graduated high school were also more likely to have poor health knowledge (2.08; 1.05–4.14). Regarding current alcohol use, as compared with non/light drinkers, poor health knowledge was more likely among moderate drinkers (1.53; 1.12–2.09) and heavy drinkers (2.28; 1.16–4.47).

**Table 4.** Logistic regression analysis of the odds of a low score on the Japanese Health Knowledge Test (*n* = 1040)

Characteristic	Odds ratio	95% CI of odds ratio	*P*-value
Age	0.99	0.98–1.00	0.145
Sex			
Male^a^	1.00		
Female	0.91	0.66–1.25	0.557
Income (Japanese Yen)			
8 million or more^a^	1.00		
6–7.99 million	1.12	0.67–1.88	0.661
4–5.99 million	1.45	0.94–2.23	0.091
2–3.99 million	1.68	1.08–2.62	0.022
<2 million	1.84	1.02–3.31	0.042
Education			
University or graduate ​ degree	1.00		
Some college	1.26	0.76–2.08	0.366
Vocational school	1.18	0.69–2.02	0.538
High school graduate	1.43	0.99–2.06	0.058
<Grade 12	2.08	1.05–4.14	0.036
Current alcohol use			
None/light^a^	1.00		
Moderate	1.53	1.12–2.09	0.008
Heavy	2.28	1.16–4.47	0.017

^a^Reference group.

CI = confidence interval.

## DISCUSSION

Using nominal categories modeling of item response theory analysis, we developed the 15-item J-HKT for Japanese adults. The instrument had a good ability to discriminate among those with poor health knowledge. In addition, items on the J-HKT and health literacy instruments were significantly correlated. The proportion of respondents with a low score on the J-HKT was higher among those with low literacy, which provides evidence of construct validity. Further, fully 36% of the participants had poor health knowledge (defined as a score of 0–3 of a possible 15 on the J-HKT). Finally, we found that poor health knowledge was associated with low income, low educational attainment, and heavier current use of alcohol.

We used nominal categories modeling to elucidate the individual discriminating power and the effect of item position in the initial 48-item pool. This allowed us to identify items with good psychometric characteristics for inclusion in the 15-item J-HKT. Therefore, it is likely that we successfully developed a test that performs well in assessing health knowledge level among people with moderately poor health knowledge.

We chose to focus the discriminating capacity of this test at the low end of health knowledge, for several reasons. First, people with the lowest levels of health knowledge are those who have the worst health outcomes.^16^^,^^24^^,^^25^ An increase in health knowledge among people who already have relatively greater knowledge is desirable, but is not likely to provide the biggest health impact. Next, focusing health resources on people with poor health knowledge is a means of minimizing health disparities.^26^ People with poor health knowledge are likely to have more complex illnesses, and management of complex illnesses requires proper adherence to regimens via active patient involvement in treatment, which is more likely when illnesses are better understood.^5^

Several limitations should be noted. First, the results of our study were based on an online survey. A high proportion of Japanese adults use the internet, and while this mode of testing is much less expensive and much more convenient than in-person household interviews, it is possible that people in the sampling frame were younger, wealthier, and more educated than the general public.^27^ As such, caution should be used in extrapolating our results.

Similarly, while the participation rate in this project is satisfactory for online research, it is likely that the participants were different from nonparticipants. Different methods for sampling the general population or patient populations with experience of frequent visits to clinicians (eg, due to chronic illness) might result in different distributions of J-HKT scores. There might also have been issues related to differential item functioning between participants and nonparticipants.^27^ Although this paper presents a careful psychometric evaluation of the 15-item J-HKT, additional research is needed to ensure appropriate calibration.

Third, since this was an online survey, we do not know if the participants had help or discussed the questions with anyone else. The online panel registration system required a personal identification number and password, and did not allow participants to test more than once. However, participants had to read the questions, and poor reading skill may have resulted in an incorrect answer for an item that would have been answered correctly had it been read aloud. Further research in the form of a test-retest evaluation is needed to determine if the results of verbal administration differ from those of the written test.^28^

Fourth, based on the item information functions of the IRT analysis for each response to individual items, the content of several responses must be improved. For instance, on item 10, no participants selected responses 3 or 4, and, on item 1, nearly all participants selected response 3. Moreover, several items will require revision because of dynamic changes in the public’s awareness of health information, due to rapid turnover in health-related knowledge in this era of rapid technological advance.

In summary, the current study described an online test of health knowledge among Japanese. We carefully evaluated the psychometric properties of this test and produced an instrument that can accurately discriminate among participants with poor health knowledge. The J-HKT is a convenient and valid measure of health knowledge, and can be used for the general Japanese public. Japanese public health practitioners and clinicians can easily use this quick test for the purposes of health education and disease prevention.

## SUPPLEMENTS

### How to answer the Items below

Below are some medical terms that you may encounter or have encountered on various occasions in medical settings or situations. Please select the sentence that you think best describes each term. The objective of this test is to evaluate your awareness of healthcare terms. It is NOT a test to determine the number of correct responses. Please answer each Item based on your knowledge, even if you are unfamiliar with the terms.

**Table:**

**Item 1** Please select the sentence that best describes the term “Tumor.”	

1.	A state of cancer that can be life-threatening.
2.	Early treatment, such as surgery, is necessary because it often metastasizes throughout the body.
3.	A growth of tissue (mass of cells) that arises from abnormal cellular proliferation.
4.	Growth is slow, and it does not spread to other parts of the body or invade surrounding tissue.

**Item 2** Please select the sentence that best describes the term “Anti-tumor Drug.”	

1.	It works for all forms of cancer, so it is given to almost all cancer patients.
2.	Because this drug does not cure cancer, it is predominantly used for terminal cancer.
3.	This drug suppresses cancer cell proliferation and eliminates cancer.
4.	Due to its numerous adverse effects and limited therapeutic effect, this drug is used only when requested by patients.

**Item 3** Please select the sentence that best describes the term “Ileus.”	

1.	It has almost the same meaning as intestinal obstruction.
2.	It does not occur to people who have had abdominal surgery in the past.
3.	A condition where the passage of bowel contents is excessively rapid.
4.	A small, sac-like protrusion that develops on the intestinal wall.

**Item 4** Please select the sentence that best describes the term “Ulcer.”	

1.	Because it is benign, there is no need to worry about cancer.
2.	Duodenal ulcers may develop into cancer.
3.	A condition where the surface of mucous membrane or skin is injured and deeply gouged.
4.	Stomach ulcer usually heals on its own.

**Item 5** Please select the sentence that best describes the term “Renal Failure.”	

1.	Because it is asymptomatic and painless, treatment is generally not required.
2.	A condition where the kidney is diseased and requires or almost requires dialysis (artificial kidney).
3.	It is caused by chronic nephritis, not diabetes or hypertension.
4.	It is caused by long-term, heavy alcohol consumption and causes jaundice (yellowish pigmentation of the skin).

**Item 6** Please select the sentence that best describes the term “Influenza.”	

1.	It is what we call the “common cold.”
2.	A bacterial infectious disease caused by the influenza bacteria.
3.	It is 100% preventable by vaccine.
4.	Antibiotics are ineffective.

**Item 7** Please select the sentence that best describes the term “Arteriosclerosis.”	

1.	Changes in the artery associated with old-age.
2.	It is caused by diabetes and/or hypertension but progresses with age.
3.	It is not caused by smoking.
4.	It happens less in men than in women.

**Item 8** Please select the sentence that best describes the term “Remission.”	

1.	It is when an illness has been completely cured.
2.	It is a phenomenon in which symptoms worsen due to chronic diseases.
3.	It is when no further hospitalization or examination is necessary.
4.	It is when symptoms are gone but the illness is not completely healed.

**Item 9** Please select the sentence that best describes the term “Terminal Care.”	

1.	Medical practice that emphasizes QOL enhancement more than life-sustaining treatment.
2.	It is only for terminal cancer patients.
3.	Medical services provided at train stations.
4.	It refers to “Care of the Dying”

**Item 10** Please select the sentence that best describes the term “Hospice.”	

1.	A hospital ward where once you enter, you never leave.
2.	Hospitalization fees at a hospice cost more than fees at a regular hospital ward.
3.	Palliative care is provided to ease physical, psychological and spiritual pain of terminally-ill patients.
4.	A place for dying where no treatment is provided.

**Item 11** Please select the sentence that best describes the term “Death with Dignity.”	

1.	Administering a lethal injection for the purpose of stopping the heart and hastening death.
2.	Choosing to die peacefully and naturally, maintaining one’s dignity.
3.	Committing suicide by ingesting poison.
4.	It is when the patient refuses life-support for not wanting to cause his/her family any trouble.

**Item 12** Please select the sentence that best describes the term “Clinical Pathway.”	

1.	Comprehensive and standardized plan of care in which care categories, such as exam, surgery, administration of medication, treatment, nutrition, etc., are organized and sequenced over a specified course of time.
2.	A schedule that specifies outpatient clinic physicians based on days and specialty.
3.	An identification card required for hospital consultations.
4.	Individually-developed care schedules that emphasize each physician’s unique treatment protocol.

**Item 13** Please select the sentence that best describes the term “Metabolic Syndrome.”	

1.	An overweight person with greater-than-standard abdominal girth measurement.
2.	An obese person who has a high level of “bad” cholesterol.
3.	There is an increased risk of diabetes, hypertension, hyperlipidemia and complications due to accumulation of visceral fat.
4.	Its cause is more due to heredity than life-style habits.

**Item 14** Please select the sentence that best describes the term “EBM.”	

1.	A standard medical practice that eliminates a physician’s experience and instincts.
2.	To practice medicine based on scientific evidence but also being considerate of each patient’s situation and values.
3.	To conduct research based on assumption and imagination.
4.	To use treatment that has been reported to be effective in a small number of study cases.

**Item 15** Please select the sentence that best describes the term “Evidence.”	

1.	Treatment methods that are subjectively chosen and widely recommended by specialists.
2.	A large majority of home remedies have “evidence” and is proven effective.
3.	Treatment methods that have been proven effective in animal experiments.
4.	Scientific evidence and proof that explain the effectiveness of treatment methods and medications.
